# Cadmium suppresses the proliferation of piglet Sertoli cells and causes their DNA damage, cell apoptosis and aberrant ultrastructure

**DOI:** 10.1186/1477-7827-8-97

**Published:** 2010-08-16

**Authors:** Ming Zhang, Zuping He, Lixin Wen, Jing Wu, Liyun Yuan, Yin Lu, Chengzhi Guo, Li Zhu, Sijun Deng, Hui Yuan

**Affiliations:** 1 College of Veterinary Medicine, Hunan Agricultural University, Changsha, Hunan 410128, P. R. China; 2 Jiangxi Biotech Vocational College, Nanchang, Jiangxi 330200, P. R. China; 3 Department of Biochemistry and Molecular & Cellular Biology, Georgetown University, Medical Center, 3900 Reservoir Road NW, Washington, DC 20057, USA

## Abstract

**Objective:**

Very little information is known about the toxic effects of cadmium on somatic cells in mammalian testis. The objective of this study is to explore the toxicity of cadmium on piglet Sertoli cells.

**Methods:**

Sertoli cells were isolated from piglet testes using a two-step enzyme digestion and followed by differential plating. Piglet Sertoli cells were identified by oil red O staining and Fas ligand (FasL) expression as assayed by  immunocytochemistry and expression of transferrin and androgen binding protein by RT-PCR. Sertoli cells were cultured in DMEM/F12 supplemented with 10% fetal calf serum in the absence or presence of various concentrations of cadmium chloride, or treatment with p38 MAPK inhibitor SB202190 and with cadmium chloride exposure. Apoptotic cells in seminiferous tubules of piglets were also performed using TUNEL assay in vivo.

**Results:**

Cadmium chloride inhibited the proliferation of Piglet Sertoli cells as shown by MTT assay, and it increased malondialdehyde (MDA) but reduced superoxide dismutase (SOD) and Glutathione peroxidase (GSH-Px) activity. Inhibitor SB202190 alleviated the proliferation inhibition of cadmium on piglet Sertoli cells. Comet assay revealed that cadmium chloride caused DNA damage of Piglet Sertoli cells and resulted in cell apoptosis as assayed by flow cytometry. The in vivo study confirmed that cadmium induced cell apoptosis in seminiferous tubules of piglets. Transmission electronic microscopy showed abnormal and apoptotic ultrastructure in Piglet Sertoli cells treated with cadmium chloride compared to the control.

**Conclusion:**

cadmium has obvious adverse effects on the proliferation of piglet Sertoli cells and causes their DNA damage, cell apoptosis, and aberrant morphology. This study thus offers novel insights into the toxicology of cadmium on male reproduction.

## Background

Cadmium is used for industrial purposes all over the world and it is a ubiquitous environmental pollutant. Cadmium is regarded as a common environmental metal toxin that causes severe toxicity in various organs, including liver, kidney, bone, and testis [1-7]. Cadmium is also involved in the carcinogenesis of prostate and testicular cancer, and it is classified as a category I carcinogen in human by the International Agency for Research on Cancer [8]. Testis is a major target organ of cadmium toxicity since there is a concordance of the rises of the percentage of apoptotic testicular cells and cadmium levels in infertile men, which inversely affects sperm concentration [9].

Spermatogenesis is a cellular process by which a subpopulation of type A spermatogonia, namely spermatogonial stem cells, divide and differentiate into sperm [10,11]. Spermatogonial stem cells are maintained in specialized microenvironment called the niche that is composed of the male germ cells, somatic cells, and extracellular matrix [12]. Sertoli cells are the major somatic cells in mammalian testis. Within the seminiferous tubules, Sertoli cells lie along the basement membrane and they are in close contact with male germ cells. Sertoli cells can secret a number of growth factors, such as glial cell line-derived neurotrophic factor [13], basic fibroblast growth factor, and epidermal growth factor [14], to support the proliferation and differentiation of spermatogonial stem cells. Notably, Sertoli cells play essential roles via paracrine pathway to control all aspects of development of male germ cells in the testis, thereby regulating spermatogenesis.

Cadmium exposure is highly associated with reproductive toxicity, resulting in both animal and human male infertility. There are reports indicating that cadmium has severe toxicology in male germ cells and affects male reproduction. In rodents, it has recently been demonstrated that cadmium results in cell death of mouse and rat male germ cells [1,15] and induces mouse sperm abnormality [16]. In human, and cadmium induces fetal germ cell apoptosis [17] and adversely affect semen quality and oxidative DNA damage in human mature sperm [18]. There is a close relationship between azoospermia and serum and seminal fluid cadmium levels in infertile males [19]. In somatic cells, it has been demonstrated that cadmium causes an increase of cytoplasm of rat Sertoli cells [20]. Cadmium also induces a morphological changes of rat Sertoli cells [21] and a disruption of inter-Sertoli tight junction in rat testis [22]. In mice, cadmium exposure leads to damaged mitochondria of Sertoli cells [23]. The distribution of cadmium was observed to be enhanced in the cytoplasm of Sertoli cells in rats after cadmium exposure, suggesting that Sertoli cells are a target of cadmium toxicology [20]. However, very little information is known about the toxic effects of cadmium on somatic cells in piglet testis. This study was designated to explore the toxic effects of cadmium on piglet Sertoli cells, with focuses on the oxidative function, DNA damage, cell apoptosis and ultrastructure changes. Piglets were used in this study since pig and human have similarity in physiology, and thus the information on the toxic effects of cadmium chloride from piglet Sertoli cells may be applicable to human.

## Methods

### Procurement of piglet testes

Testes were obtained from 3-4 weeks old piglets (Commercial Farm in Changsha, Hunan, China), placed in ice-cold phosphate-buffered saline (PBS) with 600 IU/ml penicillin-streptomycin, and sent to the laboratory within 2 hours. Piglet testes were obtained as a by-product of a routine castration, and thus this study did not cause any suffering to the animals. The use of animals in this study was approved by the Institute's Ethics Committee for Care and Use of Laboratory Animals for Biomedical Research.

### Isolation, identification, and culture of piglets Sertoli cells

Testicular capsule was removed under sterile conditions, and seminiferous tubules were isolated from piglet testis using mechanical dissociation and a one step enzymatic digestion with 1 g/L collagenase and 2.5 g/L trypsin in Dulbecco's modified Eagle's medium (DMEM)/F12, pursuant to the procedure as described previously [10,24] with minor modification. Cell mixture containing male germ cells and Sertoli cells were obtained using the second enzymatic digestion using collagenase IV, hyaluronidase, and trypsin in DMEM/F12, and Sertoli cells were further separated from male germ cells by differential plating according to the procedure as described previously [25,26]. For differential plating, Sertoli cells and germ cells were placed into tissue culture dish in the DMEM/F12 supplemented with 10% fetal calf serum (FCS) for 3 hours at 34°C. Sertoli cells attached to the culture plates, whereas male germ cells remained in suspension and were removed. Cell viability of Sertoli cells was determined with 0.4% trypan blue exclusion assay.

The freshly isolated Sertoli cells were plated at a density of 2 × 10^6 ^cells/ml in DMEM/F12 supplemented with 10% FCS in a humidified incubator with 5% CO_2 _and 100% humidity for 24 hours. Sertoli cells were identified by oil red O staining and Fas ligand (FasL) expression using antibody to FasL at a 1: 100 dilution in PBS as assayed by immunocytochemistry when 80%~90% of the dish was confluent with cells. Immunocytochemical kits were purchased from Boshide Inc. (Wuhan, China). Replacement of oil red O or primary antibody with PBS was used as a negative control.

### Experimental designs and MTT assay

Five experimental groups were set up, i.e., group A, control without cadmium chloride but with DMEM/F12; group B with 10 μM cadmium chloride in DMEM/F12; group C with 20 μM cadmium chloride in DMEM/F12; group D with 40 μM cadmium chloride in DMEM/F12; and group E with 80 μM cadmium chloride in DMEM/F12.

After culture for 24 hours, the proliferation of Sertoli cells were determined using MTT assays with quadruplicate, according to the procedure as previously described [27].

For p38 MAPK inhibitor experiments, another five groups were established: group A, the control without cadmium chloride but with DMEM/F12; groups B and C with 40 and 80 μM cadmium chloride in DMEM/F12, respectively; groups D and E were incubated with 20 μM SB202190, a specific inhibitor for p38 MAPK [28], for 1 hour, and then 40 μM and 80 μM cadmium chloride were added for 24 hours respectively. MTT assays were conducted as mentioned above.

### Effects of cadmium chloride on MDA content, SOD and GSH-Px activity of piglet Sertoli cells and hepatocytes

Hepatocytes were isolated from piglet according to the procedure as we previously described [29]. Malondialdehyde (MDA) level in piglet Sertoli cells and hepatocytes was measured by the thiobarbutiric acid method [30] and was presented as nmol per mg of protein. The activity of superoxide dismutase (SOD) in Sertoli cells was measured by the xanthine oxidase method [31] and presented as units per mg of protein. Activity of Glutathione peroxidase (GSH-Px) in the sonicated Sertoli cells and hepatocytes was measured by the DTNB reaction test [32] and presented as units per mg of protein.

### Determination of cadmium chloride on DNA damage of piglet Sertoli cells

Quantitation of DNA damage in Sertoli Cells were performed using a single cell gel electrophoresis assay (comet assay) under alkaline conditions [33] with certain modifications: the time of lysis, denature, and electrophoresis was changed to 2 hours, 1 hour and 40 minutes, respectively. Sertoli cells were observed for epifluorescence under Fluophot microscope and 400 cells were counted in each group. Being excited by ultraviolet light, the nuclear DNA and the migration of DNA in orange-red DNA image (the comet tail) was clearly observed. The rate of DNA tail was used to show the degree of DNA damage including grade 0 to 4 [34,35].

### Flow cytometric analysis of cadmium chloride on apoptosis of piglet Sertoli cells

The Annexin V-FITC Kit can be used to detect apoptotic, necrotic, and dead cells through the specific binding of FITC-labeled Annexin V to phosphotidylserine. Piglet Sertoli cells without or with various concentrations of cadmium chloride were collected and washed in PBS. Apoptosis rate of piglet Sertoli cells were detected using flow cytometry pursuant to the Annexin V-FITC/PI Kit (Nanjing Kaiji Biological Inc., China) instruction.

### Effects of cadmium chloride in piglet testis in vivo

Sixteen piglets (3 to 4 weeks) were randomly classified into four groups. Each group was intraperitoneally injected with cadmium chloride 0, 300 mg/kg body weight, 600 mg/kg, 1200 mg/kg once a day, respectively. After treatment for 6 days, animals were sacrificed, and paraffin-embedded testis sections were prepared based on the procedure as previously described [36]. Sertoli cell apoptosis was detected according to instruction of TUNEL assay kit (Boshide Inc., Wuhan, China).

### RT-PCR analysis of mRNA expression of transferrin and androgen binding protein

Total RNA was extracted from Sertoli cells without or with various concentrations of cadmium chloride using Trizol reagent (Invitrogen) and reverse transcribed into the first-strand cDNA in 20 μl of reaction primed by oligo(dT)12-18 primer using the Superscript II reverse transcriptase according to the procedure [10]. The first-strand cDNA was used as template for the PCR reactions using Taq DNA polymerase. The PCR reaction was performed as follows: transferrin: 94°C for 4 min; 94°C for 30 s, 52°C for 30 s, 72°C for 30 s, 30 cycles; 72°C extended for 7 min. Androgen binding protein: 94°C for 4 min; 94°C for 30 s, 55°C for 30 s, 72°C for 30 s, 30 cycles; 72°C extended for 7 min. β-actin: 94°C for 4 min; 94°C for 30 s, 51°C for 30 s, 72°C for 30 s, 30 cycles; 72°C extended for 7 min. Primer were synthesized by Shanghai Biological Engineering Company, and their sequences were listed as follows: transferrin, forward: 5'-GCAGTGGCCAGTTTCTTCTC-3', reverse: 5'-TTAAACAGCAGGTCCTTCCC-3', PCR product size: 458 bp; androgen binding protein, forward: 5'-GTGGATGGGAAGGAGCTGC-3', reverse: 5'-CTTGGAGACTGAATTCTGCTG-3', PCR product size: 283 bp; β-actin, forward: 5'-TTGTAACCAACTGGGACGATATGG-3' reverse: 5'-GATCTTGATCTTCATGGTGCTAG-3'(PCR product size: 763 bp). PCR products were separated by electrophoresis on agarose gels, and densitometric analyses were processed with gray image analyzer.

### Transmission election microscopy

The ultrastructural features of piglet Sertoli cells without or with various concentrations of cadmium chloride were determined under transmission election microscopy according to the procedure as described previously [29].

### Statistical analysis

All values were presented as mean ± SEM, and statistically significant differences (p < 0.05) and extremely significant differences (p < 0.01) among cadmium chloride-treated groups and the control were determined among various groups by ANOVA and Tukey post-test using SPSS 12.0 statistical software.

## Results

### Isolation, identification, and culture of piglet Sertoli cells

Piglet Sertoli cells were isolated by a 2-step enzymatic digestion and followed by differential plating. Cell viability was up to 90% as assayed by trypan blue exclusion. After 24 hours of culture, Sertoli cells attached to the dish and assumed a large columnar or irregular appearance with an elongated cellular body. Cells were connecting with each other showing mosaic-like arrangement and intercellular irregular protrusions. At the two poles of cells, there were several prominences and strong refraction. Phagocytosed objects, various sizes of vacuoles, and the cell nucleus were observed in the cytoplasm of the cells.

**Figure 1:**
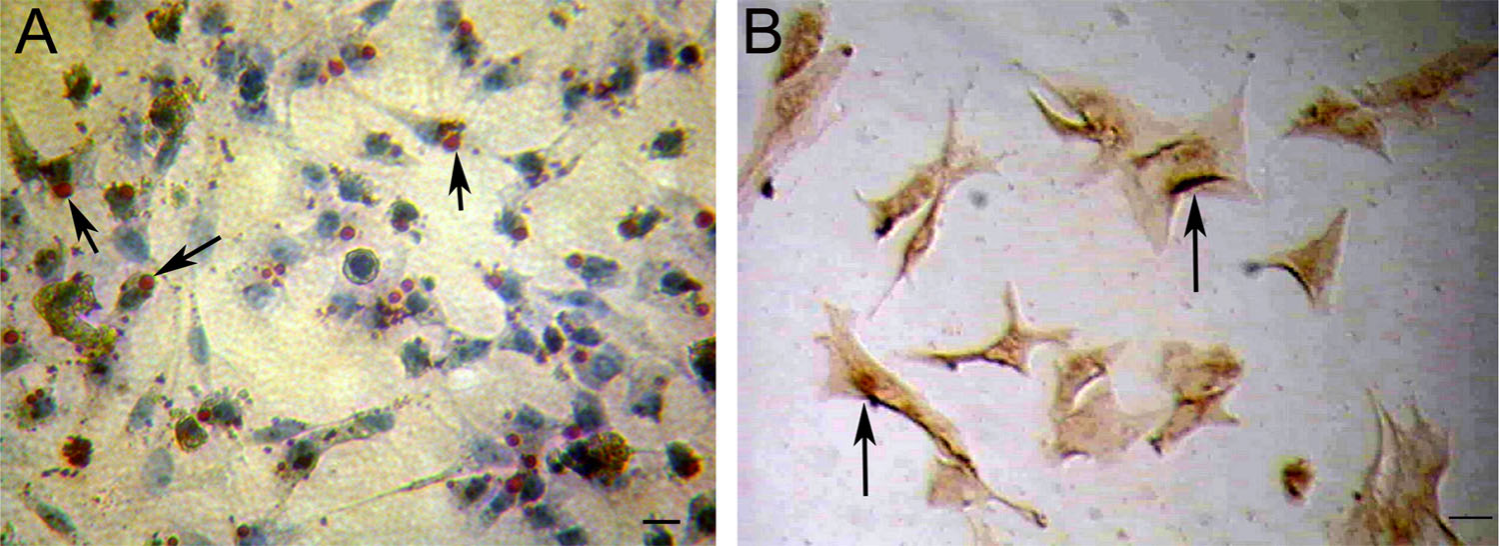
**Identification of the isolated piglet Sertoli cells**. **A:** Oil red O staining showed that red lipid droplets (arrows) were presented near the nucleus or at the two poles of cytoplasm of the isolated piglet cells, which confirmed the identity of piglet Sertoli cells. Cell nuclei were counterstained with hematoxylin. **B: **Immunocytochemistry revealed that the isolated cells were positive for FasL (arrows), further verifying the identity of piglet Sertoli cells. Scale bars in A and B = 10 μm.

Oil red O staining showed that red lipid droplets occurred near the nucleus or at the two poles of cytoplasm (Fig. 1A). No staining was observed when oil red O was replaced with PBS (data not shown). High level expression of FasL was seen in the cells as shown by immunocytochemical analysis (Fig. 1B). No staining was observed when primary antibody was replaced with PBS (data not shown). Oil red O staining can be used to detect the lipid droplets in Sertoli cells, and it has been used as a criterion to evaluate phagocytic ability of Sertoli cells [37]. FasL is a hallmark for Sertoli cells [38]. Oil red O and FasL staining was not seen in isolated Leydig cells or male germ cells (data not shown). Thus, our results verified that the isolated cells were indeed Sertoli cells. The purity of isolated Sertoli cells was more than 95% as assayed by immunocytochemical staining with oil red O and FasL.

### Effects of cadmium on piglet Sertoli cell's proliferation

**Figure 2:**
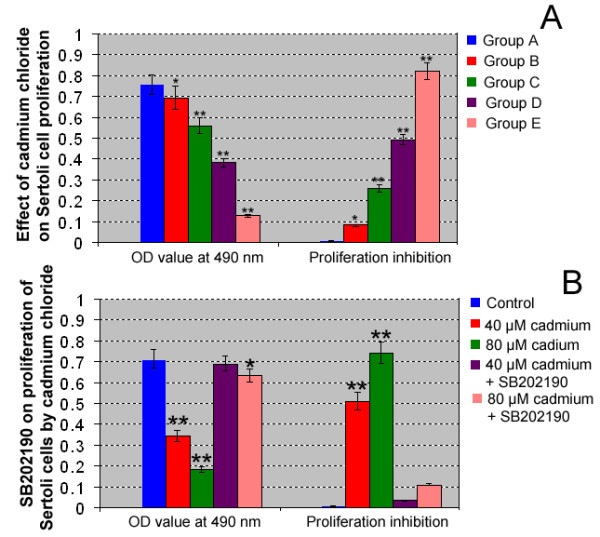
**Effect of cadmium chloride and p38 MAPK inhibitor SB202190 on proliferation of piglet Sertoli cells**. **A:** MTT assay showed cadmium chloride caused the proliferation inhibition of piglet Sertoli cells. **B: **MTT assay revealed that p38 MAPK inhibitor SB202190 alleviated the proliferation inhibition of piglet Sertoli cells caused by cadmium chloride. Compared to control group A, "*" indicated significant difference (p < 0.05), "**" indicated extremely significant difference (p < 0.01).

Cadmium is a high metal toxin that affects a variety of cellular events, including proliferation and survival. We first probed whether cadmium had an adverse effect on the proliferation of piglet Sertoli cells. After 24 hours' incubation of piglet Sertoli cells with different concentrations of cadmium chloride, the effect on their proliferation was assessed by MTT assay. As shown in Fig. 2A, Sertoli cells' proliferation decreased gradually with the increased concentration of cadmium chloride. Cell growth inhibition was significantly different (p < 0.05) in group B and extremely significantly different (p < 0.01) in groups C, D and E when compared to control group A. Moreover, the results showed that cadmium chloride inversely affected the growth of Sertoli cells in a dose-dependent manner. Notably, the inhibition rates of groups D and E treated with p38 MAPK inhibitor SB202190 decreased significantly compared with group B and C without SB202190 treatment (Fig. 2B).

### Effects of cadmium on antioxidant enzymes activities of piglet Sertoli cells

**Figure 3:**
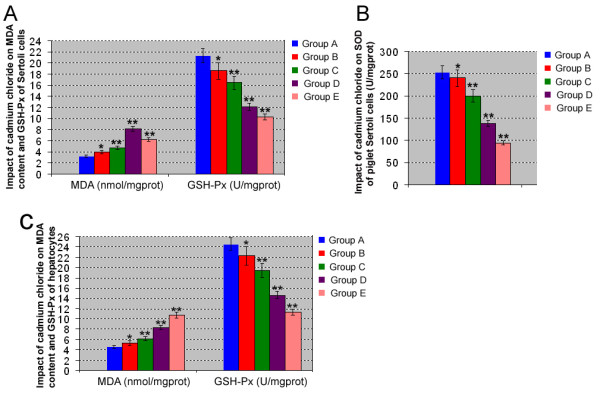
**Effect of cadmium chloride on MDA content, GSH-Px, and SOD of piglet Sertoli cells and hepatocytes**. **A-B: **Exposure of cadmium chloride to piglet Sertoli cells resulted in an increase of MDA content as well as a decrease of GSH-Px and SOD. **C: **Exposure of cadmium chloride to piglet hepatocytes led to an increase of MDA content and a decrease of GSH-Px. Compared to control group A, "*" indicated significant difference (p < 0.05), "**" indicated extremely significant difference (p < 0.01).

MDA is regarded as a major marker of lipid peroxidation in tissue, while SOD and GSH-Px are two important enzymes in the antioxidant defense system. After exposure to cadmium chloride, MDA content, SOD, and GSH-Px activities of Sertoli cells were measured and the data was summarized in Fig. 3. Compared with control group A, an increase of MDA content in group B was observed (Fig. 3A). Notably, the increase of MDA content in groups C, D and E was extremely significant (p < 0.01) compared to control group A. These results indicate that the increase of MDA content in Sertoli cells by cadmium is also dose-dependent, although MDA content in group E was lower than that in group D. In direct contrast, the SOD and GSH-Px activities in group B were found to be significantly decreased when compared to control group A, while a marked decline in SOD and GSH-Px activities was observed (p < 0.01) in groups C, D and E (Fig. 3A and 3B). The decrease levels in SOD and GSH-Px activities were closely associated with the concentration of cadmium chloride.

After treatment with cadmium chloride for 24 h, MDA content and GSH-Px activity of hepatocytes were determined. As shown in Fig. 3C, an increase of MDA content in cadmium-treated groups was observed when compared to the control A. In contrast, GSH-Px activities in cadmium chloride-treated groups decreased in comparison with control in a dose-response relationship. These results suggest that cadmium caused changes of antioxidant enzymes activities of both piglet Sertoli cells and hepatocytes.

### Effects of cadmium on DNA damage of piglet Sertoli cells

**Figure 4:**
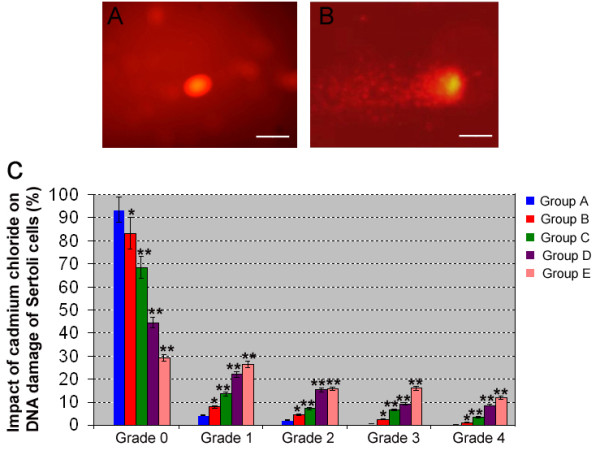
**The single-cell gel electrophoresis (comet assay) showed the DNA damage of cadmium chloride on piglet Sertoli cells**. **A: **DNA image of control group A. **B: **Comet assay revealed that the damaged DNA of cadmium chloride-treated group B contained strand breaks and migrated farther in the gel than intact DNA, creating an image resembling a celestial comet. Scale bars in A and B = 10 μm. **C: **Quantitation assay showed that DNA damage grades of piglet Sertoli cells by various concentration of cadmium chloride. Compared to control group A, "*" indicated significant difference (p < 0.05), "**" indicated extremely significant difference (p < 0.01).

Comet tail length is an important parameter in evaluating the DNA damage. After UV excitation, Sertoli cell DNA from each group showed an orange-red color. Typical and clear images of nuclear DNA and migration of DNA (tailing DNA) were observed in cadmium chloride-treated groups (Fig. 4B), whereas there is no tailing DNA in control group (Fig. 4A). As seen in Fig. 4C, the degree of DNA damage in control did not reach to grade 3 or 4, whereas DNA damage at grade 0 comprised 93.56%. With the rise of cadmium concentration, an increase of DNA tailing rate was observed. These results suggest that cadmium chloride induces DNA damage in Sertoli cells. Furthermore, we found that the overall level of DNA damage gradually increased with cadmium concentration in an obvious dose-response manner.

### Effects of cadmium on apoptosis of piglet Sertoli cells

**Figure 5:**
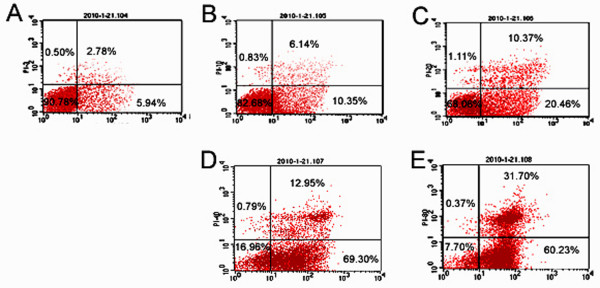
**Flow cytometry showed the apoptosis of piglet Sertoli cells without or with various concentration of cadmium chloride exposure**. **A: **Control group A; **B: **Group B; **C: **Group C; **D: **Group D; and **E: **Group E.

We next asked whether cadmium cause apoptosis of piglet Sertoli cells. By conjugating FITC to Annexin V it is feasible to identify and quantitate apoptotic cells on a single-cell basis using flow cytometry. Staining cells simultaneously with FITC-Annexin V (green fluorescence) and the non-vital dye propidium iodide (red fluorescence) allows the discrimination of intact cells (FITC^-^PI^-^), early apoptotic (FITC^+^PI^-^) and late apoptotic or necrotic cells (FITC^+^PI^+^). In the flow cytometry two-parameter dot plot: the left bottom quadrant shows viable cells, right bottom quadrant for early apoptotic cells, right upper quadrant for late apoptotic cells, while the left upper quadrant for non-living cells, namely necrotic cells. As shown in Fig. 5, the apoptosis of Sertoli cells increased gradually with the increasing of cadmium concentration. The apoptosis rate in group B was 10.35%, which was not significant difference compared with control, whereas apoptosis rate in group C, D, and E were significantly increased compared to control group A, and it showed a dose-dependent manner. Sertoli cells in low and median concentration of cadmium were mainly on early stage of apoptosis, while high concentration group of cadmium showed late stage of apoptotic cells.

### Effects of cadmium on Sertoli cells apoptosis detected by TUNEL assay in vivo

**Figure 6:**
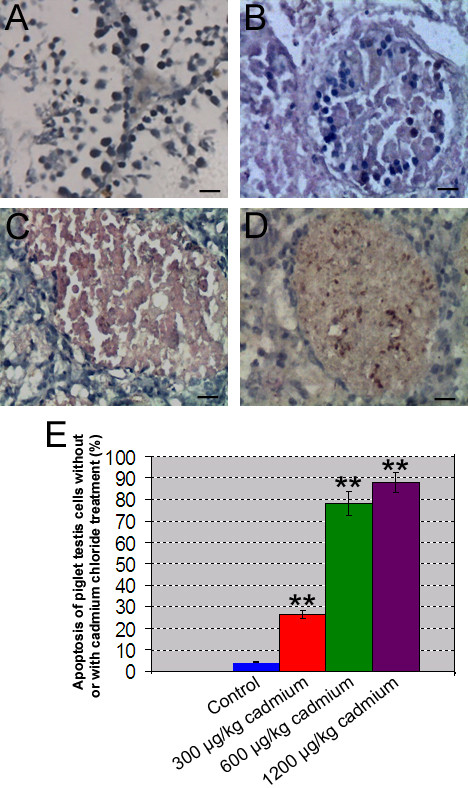
**TUNEL assay showed apoptotic cells in the seminiferous tubules of piglets with different doses of cadmium chloride treatment**. **A-D: **TUNEL-positive cells in the seminiferous tubules of piglets without cadmium chloride treatment **(A)**, or treated with cadmium chloride at 300 mg/kg body weight **(B)**, 600 mg/kg body weight **(C)**, or with 1200 mg/kg body weight **(D)**. **E: **Quantitation assay showed that TUNEL-positive cells in the seminiferous tubules of piglets treated with various concentration of cadmium chloride. Compared to control group A, "*" indicated significant difference (p < 0.05), "**" indicated extremely significant difference (p < 0.01).

Piglets were exposed to cadmium chloride with different doses for 6 days. Later stage of cell apoptosis was observed using TUNEL assay. Fig. 6 showed TUNEL-positive cells in the seminiferous tubules of piglets. High doses of cadmium led to massive Sertoli cell necrosis and more TUNEL-positive cells (Fig. 6C, D, and 6E) compared to the control (Fig. 6A) and lower dose of cadmium (Fig. 6B). These data is consistent with our in vitro study showing that cadmium induced DNA damage of Sertoli cells (Fig. 4).

### Effects of cadmium on transferrin and androgen binding protein mRNA expression

**Figure 7:**
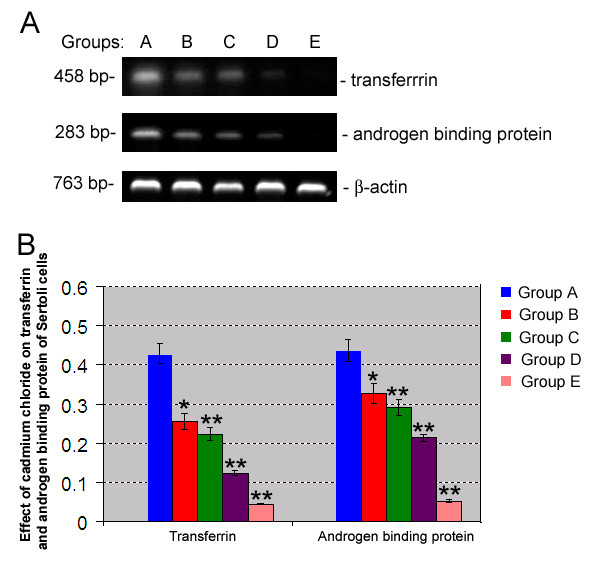
**Effect of cadmium chloride on mRNA expression of transferrin and androgen binding protein of piglet Sertoli cells**. **A: **RT-PCR showed the mRNA expression of transferrin and androgen binding protein on piglet Sertoli cells treated with various concentration of cadmium chloride. **B: **Quantitation assay showed mRNA expression of transferrin and androgen binding protein of piglet Sertoli cells by various concentration of cadmium chloride. Compared to control group A, "*" indicated significant difference (p < 0.05), "**" indicated extremely significant difference (p < 0.01).

Transferrin is a marker for Sertoli cells and androgen binding protein is a functional symbol for Sertoli cells [39,40]. We further detected transferrin and androgen binding protein gene expression in piglet Sertoli cells with or without cadmium exposure. RT-PCR revealed that mRNA expression of transferrin and androgen binding protein was expressed at high levels in control, suggesting that the isolated cells are phenotypically Sertoli cells. Of note, the expression of transferrin and androgen binding protein was suppressed by cadmium and decreased to be almost undetected in group E (Fig. 7). These results implicate that exposure of cadmium to Sertoli cells leads to a significant inhibition in the transcription of transferrin and androgen binding protein.

### Effects of cadmium on ultrastructure of piglet Sertoli cells

**Figure 8:**
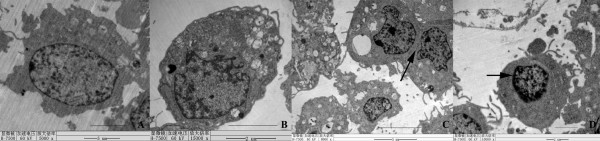
**Transmission election microscopy revealed ultrastructure of piglet Sertoli cells without or with various concentration of cadmium chloride exposure**. Note: **A: **Control group A; **B: **Group B; **C: **Group D. Arrow indicated nucleus that were dissolved into several nuclear dense bodies with condensed chromatin. **D: **Group E. Arrow indicated apoptotic bodies. Scale bars in A and C = 5 μm; bars in B and D = 2 μm.

We further assessed whether cadmium has an adverse impact on the ultrastructure of Sertoli cells using transmission electronic microscopy. Sertoli cells of control group A had intact organelles, including mitochondria and their cristae in the cytoplasm, well-developed nucleolus, and steady nuclear chromatin and less heterochromatin (Fig. 8A). In Groups B and C, a series of abnormal morphological characteristics were observed: 1) nuclei were significantly increased and they were in irregular shapes; 2) organelles were condensed; 3) nuclear chromatins forming crescent-shapes were distributed along the inner nuclear membrane; 4) some apoptotic cells had no obvious characteristics in nucleus, and a large number of vacuoles in the cytoplasm, vesicle-like expanded endoplasmic reticulum, swollen mitochondria and intact cell structure were visible (Fig. 8B); 5) some apoptotic cells in group D possessed nucleus that were dissolved into several nuclear dense bodies with intact membrane and condensed chromatin (Fig. 8C); and 6) apoptotic bodies were also visible (Fig. 8D). Taken together, these results implicate that piglet Sertoli cells exposed to cadmium assume an abnormal ultrastructural features.

## Discussion

Cadmium is a ubiquitous environmental pollutant and it can affect human health through the food chain or via other approaches. As an example, heavy smoking is the commonest source human intake of cadmium that tends to accumulate in testis, and notably it has been associated with low sperm count and motility [41]. The accumulation and toxic effects of cadmium have attracted considerable attention. Previous studies have shown that testis is the target organ of cadmium and that cadmium can damage testicular tissue and reduce spermatogenesis [1,9,15-18,20]. Sertoli cells are involved in the formation of blood-testis barrier, creating a stable microenvironment, or the niche, for the development of male germ cells. More importantly, Sertoli cells play crucial roles in supporting the self-renewal and differentiation of spermatogonial stem cells into mature sperm [13,14]. Since pig share certain physiological similarity with human, we sought to explore the toxic effects of cadmium on piglet Sertoli cells, which may offer a novel insight into the toxicity of cadmium on human Sertoli cells. We found that cadmium inhibited the proliferation of piglet Sertoli cells in a dose-dependent manner, which may lead to dysfunction of Sertoli cells on spermatogenesis. Moreover, we found that inhibitory effects of cadmium chloride to Sertoli cells was obviously reduced after treatment with p38 MAPK inhibitor SB202190, confirming that cadmium signals via p38 MAPK pathway in piglet Sertoli cells and that inhibitor SB202190 is useful to block the toxicity of cadmium on Sertoli cells.

Oxidative stress is involved in the etiology of human defective sperm formation and function, thereby resulting in male infertility [42]. Superoxide dismutase (SOD) and GSH-Px, the two major part of oxidase defense system, are natural scavenger of active oxygen and superoxide anion radicals. SOD in animal blood storage is relatively high and it is against free radicals in the first line of defense [43]. SCD can also remove the body to the ultra-induced disease oxygen anions, and thus it has been regarded as a special removal agent of oxygen free radicals. SOD could remove the body of free radicals by reducing free radicals on cell structure and organization of the attacks, and it is important to defend the cells with anti-detoxification function and promote cell growth. SOD can bind to superoxide anion-specific in vivo, and can be synergistic with the GSH-Px to prevent lipid peroxidation and its metabolites on damaging body, as well as directly captures and removes free radicals such as superoxide anion [44]. GSH-Px catalyzes the reduction of hydrogen peroxide reaction, and it has a strong capacity on scavenging lipid peroxide and hydrogen peroxide induced by active oxygen and hydroxyl radical to protect biological macromolecules and membrane from hyperoxide damage. MDA is widely used to reflect the extent of lipid peroxidation in vivo, which can cause changes in cell function, genetic toxicity, DNA damage, and carcinogenesis [45,46]. We revealed that cadmium chloride reduced SOD and GSH-Px activity but it increased MDA content of piglet Sertoli cells. Our results suggest that cadmium inhibit antioxidant enzymes activity of piglet Sertoli cells in vitro. The reduction of anti-oxidative stress enzymes activity may be related to DNA damage and/or cell apoptosis of Sertoli cells and may impair the capacity of these cells against oxidative injury by decreasing their own active oxygen and lipid peroxidation, which may adversely affect spermatogenesis and male reproduction.

Kusakabe et al, using ELISA, have reported that low concentration (2.5 μM) of cadmium doesn't induce DNA damage of rat Sertoli cells [20]. We found, using the single-cell gel electrophoresis (comet assay), that cadmium with 10 μM and more can cause DNA trailing in piglet Sertoli cells. These data indicate that middle and high concentrations of cadmium can induce DNA damage of piglet Sertoli cells.

Flow cytometry can not only distinguish between apoptotic cells and necrotic cells, early apoptotic cells and late apoptotic cells, it also quantifies the apoptotic cells. We found that cadmium chloride induced apoptosis of Sertoli cells even at a low concentration of 10 μM. Moreover, with the increasing of cadmium concentration late apoptosis of Sertoli cells occurred. These results indicate that cadmium chloride can induce apoptosis of piglet Sertoli cells. This was confirmed by our morphological observations that apoptotic bodies occurred in cadmium chloride-treated piglet Sertoli cells. Additionally, under transmission election microscopy, Sertoli cells treated with cadmium chloride assumed chromatin condensation, nuclear cleaved into dense bodies, vacuoles in cytoplasm, lamellar slight endoplasmic reticulum expansion, swelling mitochondria, and pathological vacuoles. Abnormal changes of mitochondria and endoplasmic reticulum in Sertoli cells suggest cadmium affect mitochondria via respiratory chain [47]. Mitochondria play an important role in cell proliferation and metabolism. Thus, ultrastructure changes in mitochondria would impair its function of Sertoli cells, thereby adversely affecting spermatogenesis [6,48].

## Conclusions

In summary, we have demonstrated for the first time that cadmium has obvious toxic effects on piglet Sertoli cells, as evidenced by the inhibition of Sertoli cell proliferation, DNA damage, cell apoptosis, and aberrant morphology of piglet Sertoli cells. This study thus provides novel insights into the toxicology of cadmium on male reproduction.

## Lists of abbreviations

DMEM: Dulbecco's modified Eagle's medium; FasL: Fas ligand; FCS: fetal calf serum; GSH-Px: Glutathione peroxidase; MDA: malondialdehyde; SOD: superoxide dismutase.

## Completing interest

The authors declare that they have no conflict of interests.

## Authors' contributions

MZ, ZH and HY were responsible for designing and coordinating the study as well as for data interpretation and writing of the manuscript. MZ, LW, JW, LY, YL, CG, LZ, and SD performed the experiments. MZ, ZH, and HY were involved in data collection and data analysis of the study. All authors read and approved the final manuscript.
